# Hippocampal CA2 Organizes CA1 Slow and Fast γ Oscillations during Novel Social and Object Interaction

**DOI:** 10.1523/ENEURO.0084-20.2020

**Published:** 2020-03-31

**Authors:** Logan Y. Brown, Georgia M. Alexander, Jesse Cushman, Serena M. Dudek

**Affiliations:** 1 Neurobiology Laboratory, National Institute of Environmental Health Sciences, National Institutes of Health, Research Triangle Park, NC 27709; 2 Neuroscience Curriculum, University of North Carolina at Chapel Hill, University of North Carolina School of Medicine, Chapel Hill, NC 27599

**Keywords:** CA2, DREADD, hippocampus, oriens, slow γ, social

## Abstract

A key goal in hippocampal research is to understand how neuronal activity is generated and organized across hippocampal subregions to enable memory formation and retrieval. Neuronal activity in CA2 is regulated by spatial and social investigation as well as by novelty (Mankin et al., 2015; Alexander et al., 2016), and CA2 activity controls population oscillatory activity in the slow γ and ripple ranges within hippocampus (Kay et al., 2016; Oliva et al., 2016; Boehringer et al., 2017; Alexander et al., 2018). CA2 neurons are also required for social recognition memory (Stevenson and Caldwell, 2012; Hitti and Siegelbaum, 2014; Smith et al., 2016). Because CA1 exhibits layer-specific organization (Scheffer-Teixeira et al., 2012; Lasztóczi and Klausberger, 2014, 2016) reflective of its inputs (Fernández-Ruiz et al., 2012; Schomburg et al., 2014), and because CA2 activity controls CA1 slow γ (Alexander et al., 2018), we hypothesized that silencing CA2 would affect CA1 slow γ in a layer-specific manner during investigation of a novel social stimulus. While recording from CA1, we leveraged molecular tools to selectively target and inhibit CA2 pyramidal cells using inhibitory DREADDs while subject mice investigated novel animals or objects. We found that CA2 inhibition reduced slow γ power during investigation of a novel animal and fast γ power during both novel object and animal investigation in a manner reflective of the CA2 axonal projection zones within CA1. Our results suggest that CA2 contributes to CA1 slow and fast γ oscillations in a stimulus-specific manner.

## Significance Statement

Using chemogenetics and electrophysiology, we show that CA2 contributes to slow γ oscillations in CA1 during novel animal investigation and fast γ during novel animal or object investigation in a layer-specific manner reflective of CA2 connectivity. Slow γ oscillations in CA1 rely on CA3 and CA2 input during running, and CA2 neuronal activity is required for social memory. Our findings of reduced slow γ oscillations in CA1 layers targeted by CA2 axons on reduction of CA2 activity support the involvement of CA2 in slow γ oscillations, and the finding that slow γ oscillations were impaired by CA2 silencing selectively during novel animal investigation are consistent with a role for CA2 in social cognition.

## Introduction

The mammalian hippocampus is critical for the formation of long-term episodic memories (Tulving, 1984). Despite the relative stability of neuronal connections post-development, neurons may be capable of functionally coupling and uncoupling to one another based on their frequency characteristics (Buzsáki and Draguhn, 2004). Although oscillations detected in the local field potential (LFP) are highly structured across brain areas, the precise patterns of synchronization change dynamically with behavior.

Rodent hippocampal oscillations are dominated by three frequencies of activity: theta (∼6–9 Hz; Buzsáki et al., 2003), γ (∼25–100 Hz; Colgin, 2016), and ripples (∼100–200 Hz; Csicsvari et al., 1999). γ Can be further divided into slow (∼25–55 Hz) and fast (∼55–100 Hz) bands. These two frequencies are thought to be generated through separate mechanisms and play distinct roles in memory (Colgin et al., 2009). Slow γ in CA1 is prevalent in stratum radiatum (SR; Schomburg et al., 2014), where CA3 neuronal projections predominate, and are coherent with slow γ in CA3 (Colgin, 2015). Further, silencing of CA3 synaptic output with tetanus toxin light chain reduces slow γ power in CA1 by ∼30% (Middleton and McHugh, 2016). CA2 has also been shown to contribute to slow γ in CA1, as chemogenetic manipulation of CA2 activity bi-directionally modulates CA1 slow γ power during running (Alexander et al., 2018). In contrast, fast γ oscillations are prevalent in stratum lacunosum moleculare (SLM) of CA1 (Schomburg et al., 2014), co-occur with fast γ in medial EC (Colgin et al., 2009), and are impaired by lesioning of medial EC (Bragin et al., 1995), supporting the notion that CA1 fast γ relies on medial EC activity. Fast γ is thought to contribute to memory encoding because fast γ power is increased there during an animal’s investigation of novel stimuli (Kemere et al., 2013; Zheng et al., 2016).

Pyramidal neurons in area CA2 strongly express a number of genes, including *Amigo2* (Lein et al., 2007; Laeremans et al., 2013), *PCP4* (San Antonio et al., 2014), *RGS14* (Lee et al., 2010), and others (Dudek et al., 2016). Neurons in CA2 receive excitatory input from the EC and CA3 (Chevaleyre and Siegelbaum, 2010; Kohara et al., 2014) and, in turn, project to a variety of intra- and extra-hippocampal targets, but primarily target CA1 stratum oriens (SO) and deep pyramidal cells (Tamamaki et al., 1988; Kohara et al., 2014). Neurons in CA2 also project to CA1 SR to a lesser extent, but not to SLM. Several studies have investigated the function of CA2 *in vivo*, implicating it in the organization of sharp wave ripple (Kay et al., 2016; Oliva et al., 2016) and slow γ oscillations (Alexander et al., 2018), as well as social memory (Stevenson and Caldwell, 2012; Hitti and Siegelbaum, 2014; Alexander et al., 2016; Smith et al., 2016). However, it currently remains unknown whether CA2 coordinates hippocampal oscillations during social behaviors.

Given the evidence for CA2’s role in social memory and that CA1 γ oscillations are regulated in a layer-specific manner reflective of its upstream inputs (Fernández-Ruiz et al., 2012; Scheffer-Teixeira et al., 2012; Lasztóczi and Klausberger, 2014, 2016), we predicted that if CA2-dependent oscillations in CA1 contribute to its function in social memory, then they should occur during social exposure and, given the distribution of CA2 axons in CA1, should be organized in a layer-specific manner. Thus, we asked whether CA1 γ oscillations also rely on CA2 output during investigation of novel social stimuli. We recorded from each layer of CA1 in adult, male *Amigo2*-icreERT2 mice while mice investigated a novel social or object stimulus and then inhibited CA2 pyramidal cells using human-muscarinic-4-DREADD-Gi (hM4Di) to test whether CA2 contributes to γ oscillations in CA1 during novel object and social stimulation.

## Materials and Methods

### Animals

Experiments were conducted in adult male (more than eight-week-old) *Amigo2*-icreERT2 mice. Mice were housed under a 12/12 h light/dark cycle with *ad libitum* access to food and water. Mice were naive to any treatment, procedure or testing at the beginning of the experiments described here. Mice were group-housed until the time of virus infusion, at which point they were singly housed. All procedures were approved by the Institute’s Animal Care and Use Committee and were in accordance with the National Institutes of Health guidelines for care and use of animals.

### Viruses

All AAV viruses were produced by the Gene Therapy Center Vector Core at the University of North Carolina at Chapel Hill and had titers of >10^12^ genome copies/ml. For chemogenetic manipulation using hM4Di-mCherry, mice were bilaterally infused with 0.5 μl of AAV5-hSyn-DIO-hM4Di-mCherry into each hemisphere of the brain.

### Stereotaxic virus infusions and tamoxifen treatment

For virus-infusion surgery, mice were anesthetized with ketamine (100 mg/kg, i.p.) and xylazine (7 mg/kg, i.p.), then placed in a stereotaxic apparatus while on a heated pad. Sedation was maintained with 1.5–2.5% isoflurane during surgery if animals regained reflex responses at any point during the surgery. Following three swabs with betadine and saline rinse, an incision was made down the midline of the scalp, a burr hole was drilled above each target region and viruses were microinjected using a 1-μl Neuros Hamilton syringe at a rate of 100 nl/min. Following infusion, the needle was left in place for five additional minutes to allow for diffusion of the virus before slowly being retracted. Injection coordinates for CA2 were −2.30 mm AP, ±2.50 mm ML, and −1.90 mm DV from bregma. The scalp was then sutured shut and animals were administered buprenorphine (0.1 mg/kg, s.c.) for pain and returned to their cages. Two weeks following AAV infusion, *Amigo2*-icreERT2 mice began daily tamoxifen intraperitoneal injections (100 mg/kg tamoxifen dissolved in warmed corn oil) for a total of 7 d. Animals were allowed to recover from tamoxifen treatment for one week before undergoing electrode implantation to allow sufficient time for estrogen receptor translocation to the nucleus and subsequent recombination.

### Electrode implantation

At least one week after the last tamoxifen treatment, mice for *in vivo* electrophysiology were implanted with NeuroNexus A1x32 silicone electrodes (NeuroNexus). Mice were anesthetized with ketamine (100 mg/kg, i.p.) and xylazine (7 mg/kg, i.p.), then placed in a stereotaxic apparatus while on a heated pad. An incision was made in the scalp, and the skull was cleaned and dried. A screw for the electrode reference wire was positioned ∼4 mm posterior and 2 mm lateral to bregma over the right cortical hemisphere and a screw for the ground wire was positioned in the skull over the cerebellum. Three additional anchor screws were secured to the skull. Electrodes were then lowered into the hole drilled over dorsal CA1: −1.30 mm AP, −1.40 mm ML, −1.85 mm DV from bregma, and the reference and ground wires were attached to their respective screws. The NeuroNexus A1x32 electrode is designed with 32 recording sites arranged linearly along a single shank, spaced 25 μm apart, providing a tissue coverage range of 775 μm.

### Recording protocol

**Figure 1.**
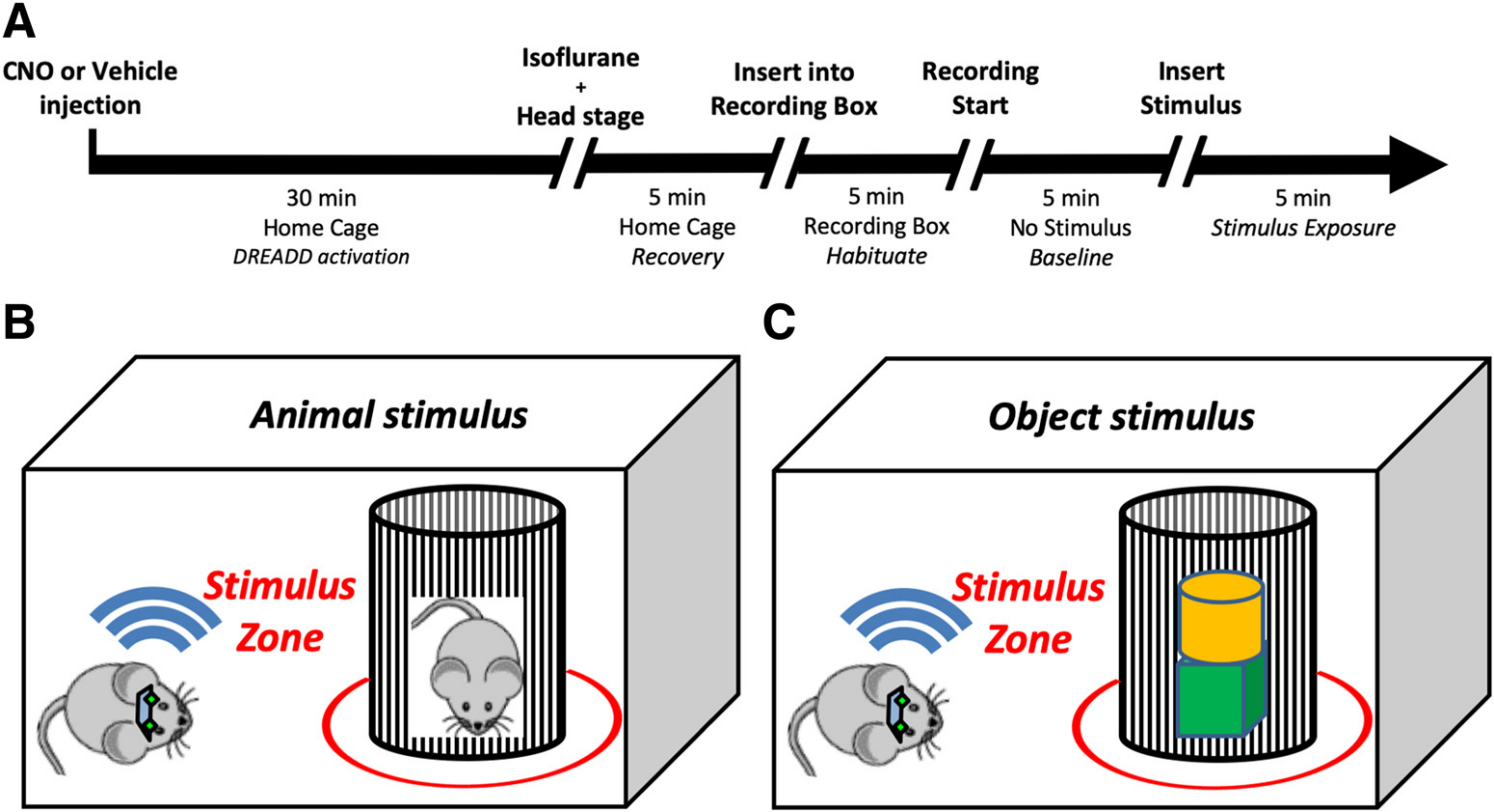
Experimental design. ***A***, Recording protocol used for each experimental recording session. ***B***, Behavioral tracking for selection of periods to analyze. A virtual 10-cm circle was drawn around the wire mesh cup and designated as the stimulus zone. Periods in which the subject’s body, nose, or tail were located within the stimulus zone were isolated and used for subsequent spectral analysis.

We recorded one session per day for eight sequential days. Treatment with clozapine-N-oxide (CNO) or vehicle alternated across days, as did the type of stimulus presented (animal or object). All recordings were made during the animal’s light phase under red light illumination to encourage exploration. For these experiments, animals were allowed to freely explore a clear Plexiglas cage (40.5 × 24.5 cm). Bedding was placed in the bottom of the cage (as in home cages) to encourage exploration. Curtains surrounded the cage to prevent viewing of the experimental room and limit light exposure. The recording protocol (Fig. 1*A*) proceeded as follows: 40 min before the start of recording, subjects were administered CNO (3.0 mg/kg) or saline and returned to their home cages outside the recording room. Ten minutes before the start of the experiments, subjects were lightly anesthetized with isoflurane to allow attachment of wireless head stages and were transferred to the recording room where room lights were turned off and red lights were illuminated. Five minutes before the start of recording, subjects were transferred from their home cage to the recording cage and allowed to habituate for 5 min before the start of recording. For the first 5 min of each recording session, no stimulus was present. This phase was used as a baseline period to assess distance traveled and time spent mobile. For the second 5 min of each recording session either a novel animal or novel object was inserted into the recording cage in a wire mesh cup. The novel animal stimulus (Fig. 1*B*) was a male, juvenile conspecific from the *Amigo2*-icreERT2 line, naive to any experimental testing before being used in the test session. The novel object stimulus (Fig. 1*C*) consisted of inanimate wooden blocks (Kaytee Pet Supplies), which varied in color and shape, measured ∼2” × 2” × 2”, and had a cedar scent. In each session, two to three blocks were stacked together and used as the object stimulus. Periods in which a subject was actively investigating the stimulus were isolated and used for spectral analysis.

### Neurophysiological data acquisition and behavioral tracking

Neural activity was transmitted via a 32-channel wireless 10× gain head stage (Triangle BioSystems International) and was acquired using the Cerebus acquisition system (Blackrock Microsystems). Continuous LFP data were bandpass filtered at 0.3–500 Hz and stored at 1000 Hz. For behavioral tracking, videos of recording sessions were acquired with a sampling rate of 30 Hz using the Neuromotive Software (Blackrock Microsystems) and an infrared camera. Behavior was analyzed offline using the Ethovision XT (Noldus) behavioral tracking software. Subjects’ smoothed instantaneous velocity, measured from reflective tape on the headstage, was used to isolate periods of running from periods of relative immobility. Samples in which the animal’s smoothed instantaneous velocity exceeded a threshold of ≥7 cm/s were designated as periods of running and excluded from analysis. The 7-cm/s threshold was selected based on manual optimization of tracking parameters to best divide periods of quiet immobile rest from active traversing of the environment. For selection of periods in which subjects were actively investigating stimuli, a virtual 10-cm circle was drawn around the wire mesh cup in which stimuli were presented, and periods in which the subject’s body, nose, or tail were located within the circle were isolated (Fig. 1*B*,*C*). For spectral analyses, only samples in which subjects were both immobile and located inside the virtual circle were used. Because the animal was not traversing the environment during the periods used for our LFP analyses, a prominent peak in the theta range was not expected. All indexed tracking information was exported from Ethovision and then used to select the appropriate LFP samples from behavioral periods of interest in MATLAB.

### Neurophysiological data analysis

All neuronal data analyses were performed with MATLAB version R2016b (MathWorks) using custom written scripts for (1) selection of LFP samples corresponding to periods in which subjects performed behaviors of interest, (2) artifact rejection, and (3) spectral analysis of LFP samples using a family of Morlet wavelets. First, behavioral vectors were generated from tracking data generated by Noldus Ethovision XT to designate periods in which subjects were both immobile and inside the virtual circle drawn around the stimulus. The behavioral vectors were then used to match periods in which animals performed behaviors of interest to the appropriate LFP samples. Second, noise and muscle artifacts were removed from recordings based on a 6-standard deviation rejection threshold; when detected, a 3-s window around the noise threshold crossing was removed. Third, spectral analysis was performed using a family of 80-wavelet carrier frequencies which increased logarithmically and had a low-frequency cutoff of 0.5 Hz and a high-frequency cutoff of 100 Hz. For each recording session, the time-frequency representation of the data were obtained by convolving the LFP with the family of wavelets to produce a vector of the same length as the original LFP data.

Over the eight recording days, each of the four-possible drug and stimulus combinations (vehicle-animal, vehicle-object, CNO-animal, CNO-object) were delivered twice, and the LFP power values from these two repeat sessions were averaged. For statistical analysis of LFP power we focused on the theta (6–9 Hz), slow γ (25–55 Hz), and fast (55–100 Hz) γ frequencies ranges. An average was calculated for each frequency band separately by averaging all wavelet values that were used to generate the time-frequency representation of that band. For example, the slow γ band was computed using 19 wavelets, so these 19 values were averaged to yield a single value for the slow γ band. These values were calculated for each hippocampal layer in each subject and used for group comparisons of power under the different experimental conditions.

**Figure 2.**
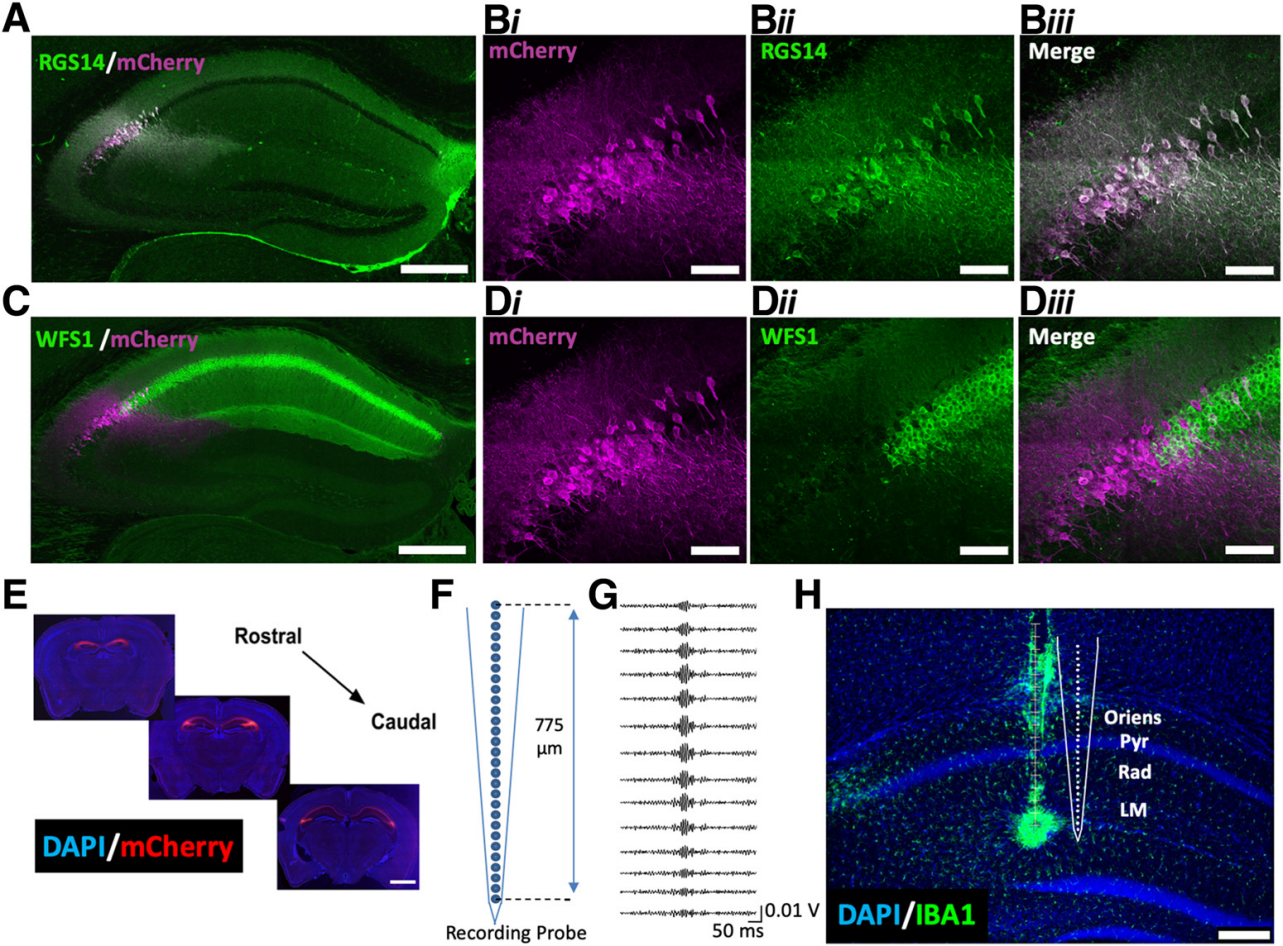
Selective targeting of CA2 for Gi-DREADD expression and CA1 for recording. *Amigo2-*icreERT2 mice were infused bilaterally with AAV-hSyn-DIO-hM4Di-mCherry, treated with tamoxifen, and implanted with NeuroNexus A1x32 silicone probes. ***A***, Co-expression of Cre-dependent hM4Di-mCherry and RGS14, a marker for CA2 pyramidal cells, in the hippocampus. ***B***, High magnification image showing the expression pattern of (***i***) hM4Di-mCherry in CA2, (***ii***) RGS14 in CA2, and (***iii***) merged images. No evidence of hM4Di-mCherry expression was found outside of the RGS14-expressing region. ***C***, Expression of Cre-dependent hM4Di-mCherry and WFS1, a marker for CA1 pyramidal cells, across the hippocampus. ***D***, High-magnification image showing the expression pattern of (***i***) hM4Di-mCherry in CA2, (***ii***) lack of WFS1 in CA2, and (***iii***) merged images. No evidence of mCherry colocalization with WFS1 was observed. ***E***, Expression pattern of hM4Di-mCherry across the rostral to caudal extent of CA2. ***F***, Schematic diagram of NeuroNexus A1x32 probe detailing probe dimensions. ***G***, Ripple-filtered (100–300 Hz) LFPs across the CA1 layers. The channel with the maximal amplitude of putative ripples was designated as located in the pyramidal cell layer. ***H***, At the end of experiments, an electrolytic lesion was made on the bottom channel of the electrode and sections were processed by IHC to highlight IBA1, a marker for activated microglia. Representative recording locations in CA1 were reconstructed based on IBA1 staining and putative ripple oscillations. Scale bars = 200 μm (***A***, ***C***, ***H***), 50 μm (***B***, ***D***), and 1 mm (***E***).

Ripple oscillations, paired with immunohistochemical (IHC) methods (described below), were used to identify electrode placement. For ripple measurements, periods in which subjects were immobile were isolated and the LFPs from these periods were filtered in the ripple range (100–300 Hz). The channel with the maximum amplitude of putative ripple events was identified as the pyramidal cell layer (Fig. 2*G*).

### IHC and electrode localization

To aid in electrode localization, at the end of all experiments for each animal, a small amount of current was passed through the channel corresponding to the bottom-most electrode pad of each electrode (5 μA for 5 s) to create a small lesion at the most ventral recording location of that probe, visible on subsequent IHC processing. Three hours after creating the lesion, animals were euthanized with Fatal Plus (sodium pentobarbital, 50 mg/ml; >100 mg/kg) and underwent transcardial perfusion with 4% paraformaldehyde. Heads with electrodes remaining implanted were submerged in 4% paraformaldehyde for 72 h. Brains were then carefully removed, rinsed in PBS, and sectioned at 40 μm on a vibratome.

For IHC, brain sections were rinsed in PBS, boiled in deionized water for 3 min, and blocked for at least 1 h in 3–5% normal goat serum/0.01% Tween 20 PBS. Sections were then incubated in mouse anti-RGS14 (UC Davis, 75170, 1:250) and rabbit anti-WFS1 (ProteinTech, 11558–1-AP, 1:250) primary antibodies. Sections in which an electrode track was visible were incubated in rabbit anti-IBA1 (Wako, catalog 012-26 723, 1:500), a marker for activated microglia (Fig. 2*H*). Antibodies were diluted in blocking solution and sections were incubated for 24 h. After several rinses in PBS/Tween, sections were either incubated in the secondary antibodies Alexa Fluor goat anti-mouse 488 (1:250, Invitrogen) and Alexa Fluor goat anti-rabbit 633 (1:250, Invitrogen) or Alexa Fluor goat anti-rabbit 488 (1:500, Invitrogen) for 2 h. Finally, sections were washed in PBS/Tween and mounted with Vectashield hardset mounting medium with DAPI (Invitrogen). Images of whole-brain sections were acquired with a slide scanner using the Aperio Scanscope FL Scanner (Leica Biosystems Inc.). The slide scanner uses a monochrome TDI line-scan camera, with a PC-controlled mercury light source to capture high resolution, seamless digital fluorescent images. Images of selected hippocampi were acquired on a Zeiss 780 meta confocal microscope using a 40× oil-immersion lens.

### Sample size and group assignment

For all experiments presented, 19 male *Amigo2*-icreERT2 (10 *Amigo2*-icreERT2+ and nine *Amigo2*-icreERT2–) mice were used. *Amigo2*-icreERT2 mice were arbitrarily selected from individual litters. No specific method of randomization was used to assign groups. Whenever possible, Cre+ and Cre– mice from the same litter were used. Animals were housed with same-sex littermates during weaning and individually housed for the duration of experiments following virus infusion. Pre-established criteria for excluding mice from analysis included (1) missed electrode implantation, (2) equipment failures during testing, and (3) insufficient investigation of experimental stimuli, with a minimum of 10 s of investigation per recorded session required to be included in the analysis.

### Statistical analyses and results reporting

**Figure 3.**
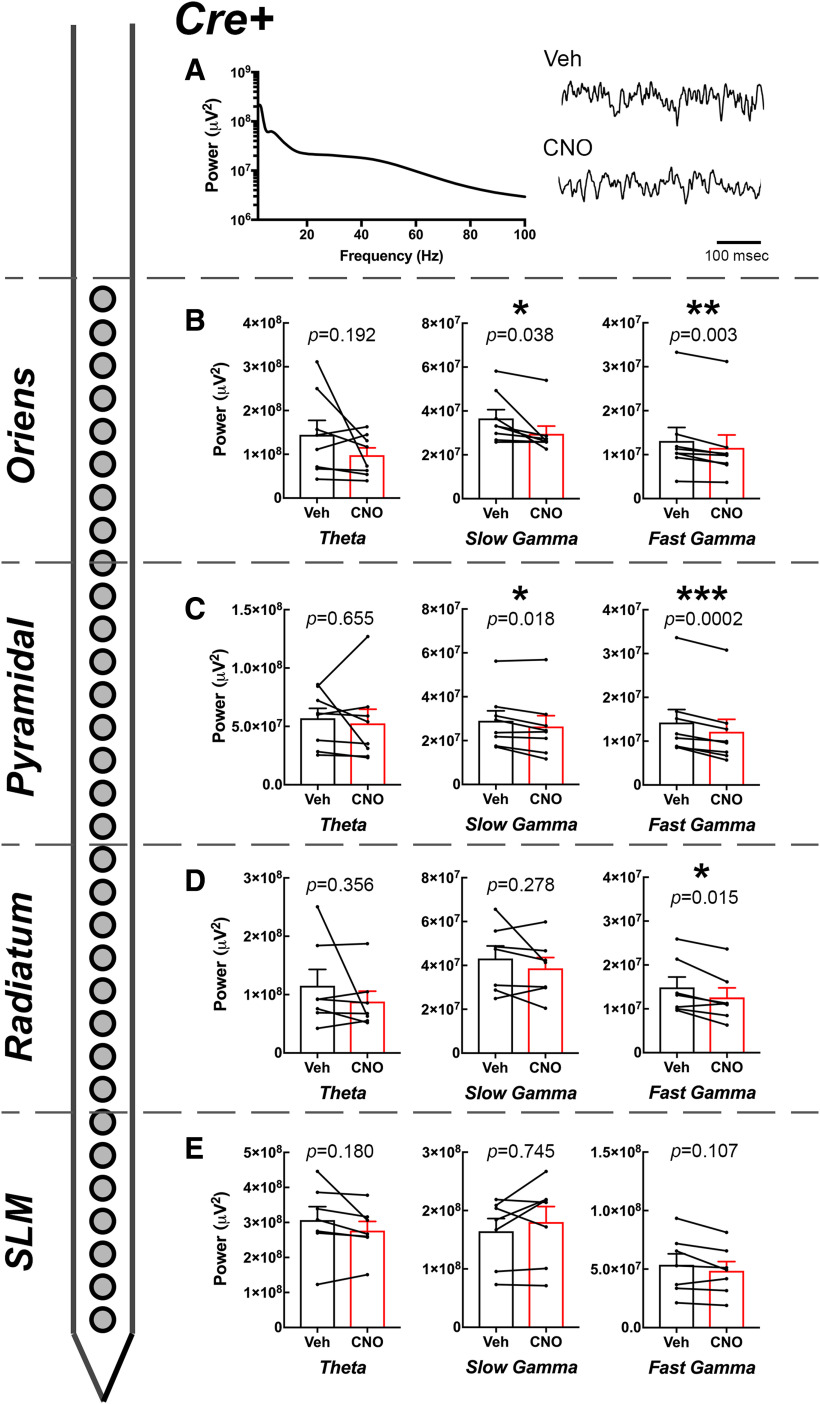
Effect of CNO on CA1 oscillatory power by layer in *Amigo2*-icreERT2+ animals. ***A***, Example PSD from the pyramidal cell layer of an *Amigo2*-icreERT2+ animal and raw LFP traces collected following either vehicle or CNO (3 mg/kg) treatment. ***B–E***, Theta, slow γ, and fast γ powers measured from each layer of CA1 as indicated by the schematized shank electrode and layer of CA1 listed on the left. For each graph, population means and SEMs are represented as colored bars (black = vehicle; red = CNO), and dots connected by lines represent data from individual animals for each treatment. Specific *p* values from paired *t* tests are shown over each graph; *p <* *0.05, **p <* *0.01, ***p <* *0.001.

**Figure 4.**
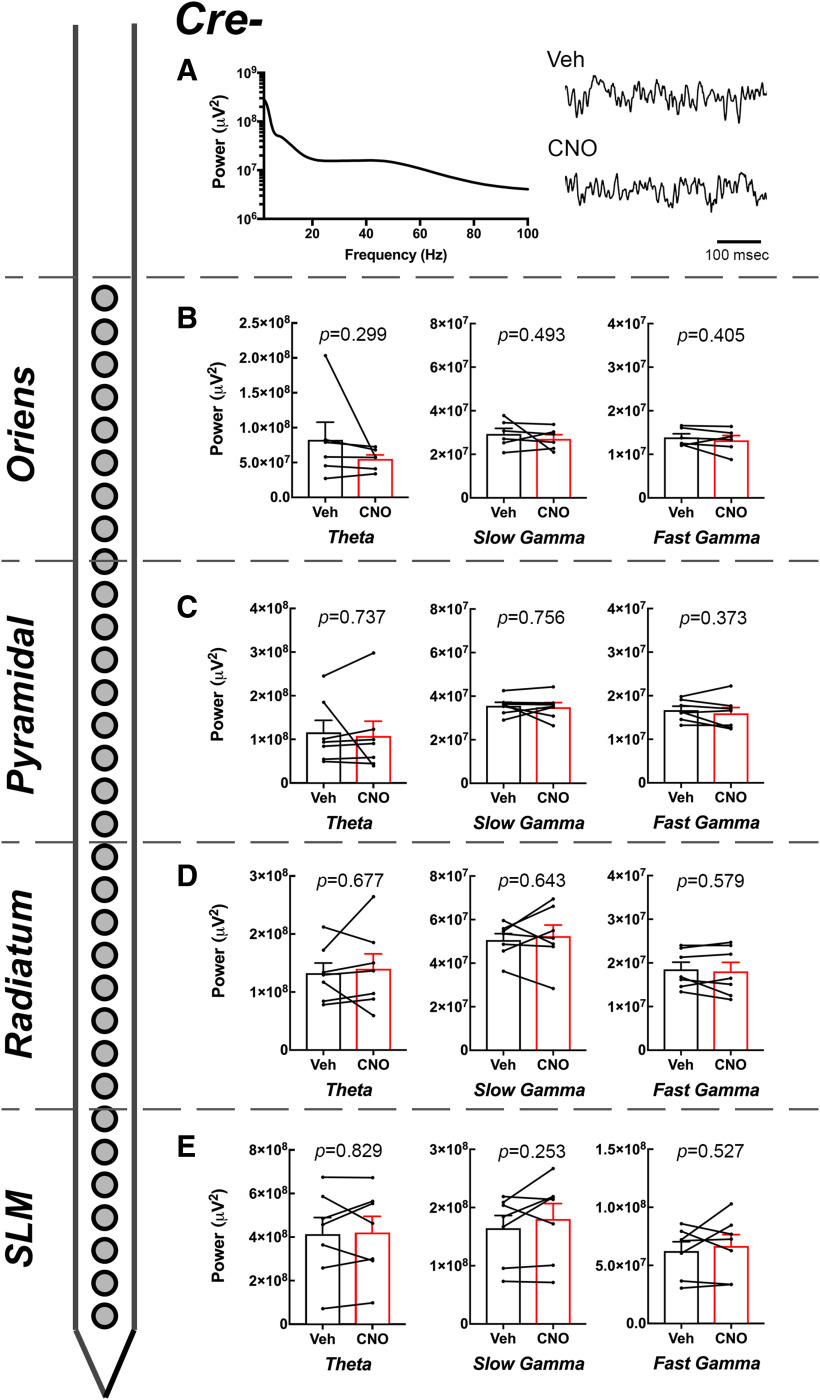
Effect of CNO on CA1 oscillatory power by layer in *Amigo2*-icreERT2– animals. ***A***, Example PSD from the pyramidal cell layer of an *Amigo2*-icreERT2– animal and raw LFP traces collected following either vehicle or CNO (3 mg/kg) treatment. ***B–E***, Theta, slow γ, and fast γ powers measured from each layer of CA1 as indicated by the schematized shank electrode and layer of CA1 listed on the left. For each graph, population means and SEMs are represented as colored bars (black = vehicle; red = CNO), and dots connected by lines represent data from individual animals for each treatment. No comparisons were significantly different between vehicle and CNO treatment, as indicated by the specific *p* values for each statistical analysis. Population means and SEMs are represented as colored bars (black = vehicle; red = CNO), and dots connected by lines represent data from individual animals for each treatment.

**Figure 5.**
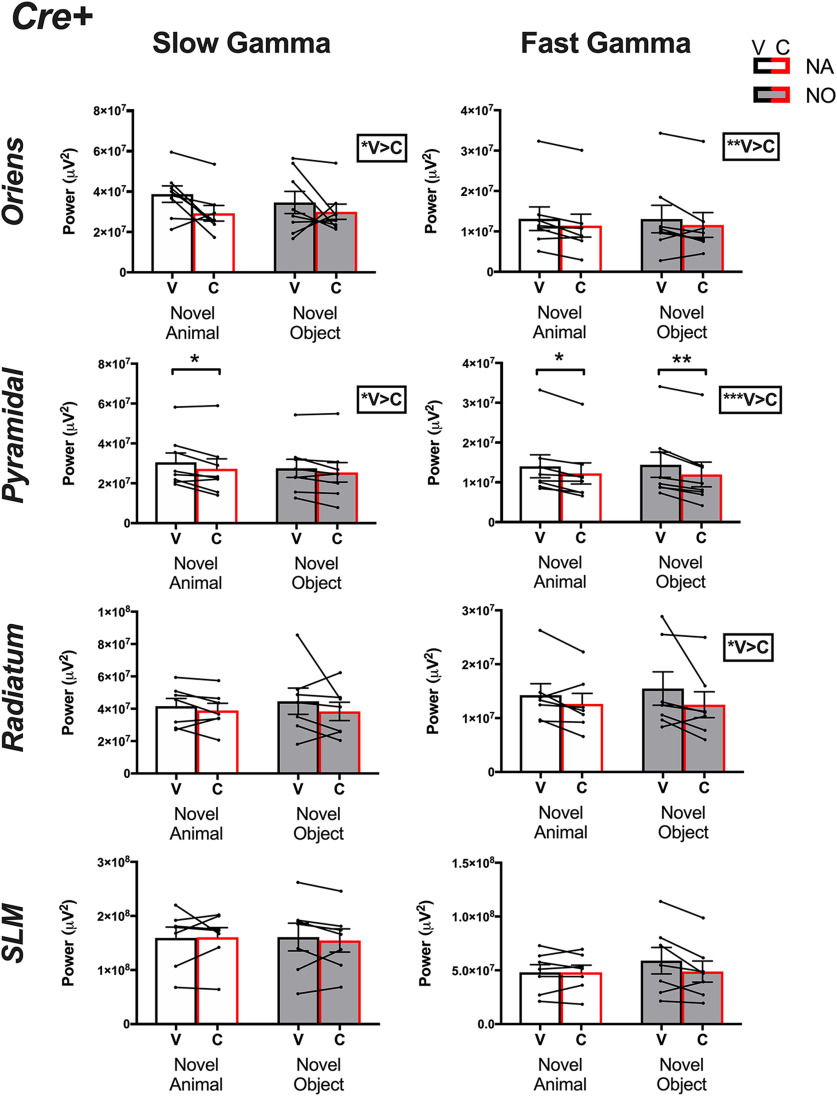
γ Powers collected from *Amigo2-icreERT2*+ animals. Slow (left column) and fast (right column) γ powers for each layer in CA1 following either vehicle (*V*, black bars) or CNO (*C*, red bars) treatment and on investigation of either a novel animal or a novel object. Two-way ANOVAs were performed within each layer and frequency. A main effect of treatment was found for slow and fast γ within each of oriens and the pyramidal cell layers as well as for fast γ in radiatum, as reported by the boxed-in result to the right of the associated graph. Population means and SEMs are represented as colored bars (black = vehicle, red = CNO and for each treatment, empty bars = novel animal, filled bars = novel object), and dots connected by lines represent data from individual animals for each treatment. Results of *post hoc* tests are shown on the graphs where significant; *p <* *0.05, **p <* *0.01, ***p <* *0.001.

**Figure 6.**
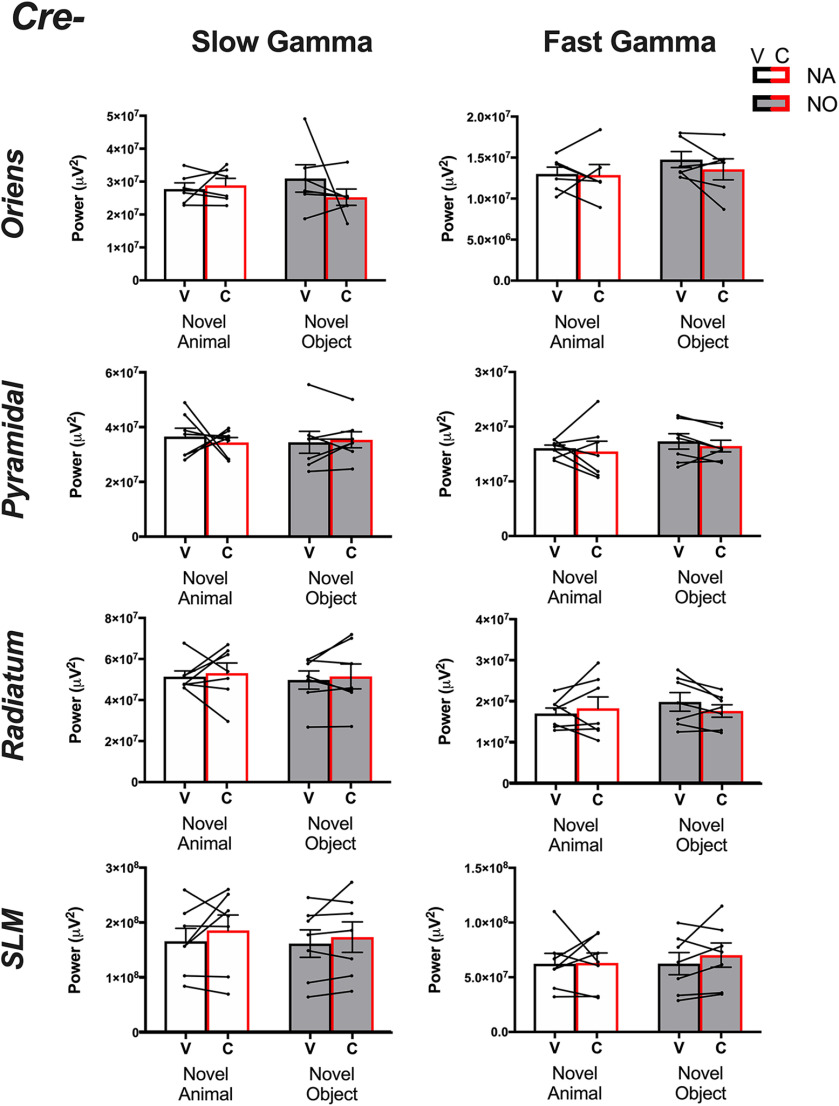
γ Powers collected from *Amigo2*-icreERT2– animals. Slow (left column) and fast (right column) γ powers for each layer in CA1 following either vehicle (*V*, black bars) or CNO (*C*, red bars) treatment and on investigation of either a novel animal or a novel object. Two-way ANOVAs were performed within each layer and frequency. No main effects of treatment were found for slow or fast γ within any layer on investigation of either stimulus. Population means and SEMs are represented as colored bars (black = vehicle, red = CNO and for each treatment, empty bars = novel animal, filled bars = novel object), and dots connected by lines represent data from individual animals for each treatment.

For each experiment presented in Results, the number of replicates is presented as *N* when indicating the number of animals that were used for the experiment. Statistical tests used for each experiment are presented in the text. Statistical significance was based on *p* = 0.05. All error bars in graphs represent SEM. For statistical analysis of γ oscillatory powers presented in Figures 3, 4, we used a repeated-measures general linear model (RM-GLM; SPSS version 21, IBM) to explore potential effects of recording layer (SO, SR, pyramidal, SLM), frequency (slow γ, fast γ), stimulus type (animal, object), and drug (vehicle, CNO). The GLM revealed a significant interaction between the layer, frequency, and drug conditions and, based on these results, we performed a series of RM, two-tailed, paired *t* tests to explore the source of the observed interaction effect. Statistical analysis of theta powers was performed separately from γ oscillatory powers due to the larger values and greater variability in theta power we observed during non-mobile investigative behaviors. Theta powers for each layer were compared between vehicle and CNO conditions using paired *t* tests. Locomotor velocity during LFP sampling and follow-up analyses of γ powers on presentation of each stimulus separately (Figs. 5, 6) were analyzed using two-way ANOVAs, with Sidak *post hoc* tests, where appropriate. For behavioral investigation time data, we used two-tailed paired *t* tests. For locomotor data, we used RM one-way ANOVAs with Geisser–Greenhouse correction for unequal variance.

## Results

### Inhibitory DREADD expression in CA2

To gain selective genetic access to molecularly-defined CA2, we used a previously-generated tamoxifen-inducible, Cre-dependent mouse line, *Amigo2*-icreERT2 (Alexander et al., 2018). Infusion of AAVs encoding Cre-dependent hM4Di (Fig. 2*A–E*) with the neuron-specific human synapsin promoter into *Amigo2-*icreERT2*+* mice allowed for selective expression of hM4Di-mCherry in CA2 pyramidal neurons without expression in other hippocampal subregions or outside of the hippocampus, as indicated by co-localization of mCherry with RGS14 (Fig. 2*A*,*B*) and lack of co-localization with WFS1 (Fig. 2*C*,*D*). mCherry expression was undetectable in control *Amigo2*-icreERT2– mice infused with hM4Di-mCherry (data not shown). This mouse line enabled us to selectively target and inhibit activity of CA2 pyramidal neurons *in vivo* and measure the resulting network and behavioral effects.

### CA1 γ oscillatory power varies by layer, frequency, and CA2 activity

Inhibition of CA2 via expression and activation of hM4Di in CA2 pyramidal cells of *Amigo2*-icreERT2*+* mice has been shown to reduce slow γ power in the pyramidal cell layer of CA1 during running (Alexander et al., 2018). Therefore, we tested whether inhibition of CA2 in the same manner would cause layer-specific changes in slow and fast γ power detected in CA1 during investigation of different stimuli. *Amigo2*-icreERT2*+* and control *Amigo2*-icreERT2– mice infused bilaterally with hM4Di were implanted with 32-channel NeuroNexus silicone probes (Fig. 2*F*). For each recording session, *Amigo2*-icreERT2*+* and control *Amigo2*-icreERT2– mice were treated with 3.0 mg/kg of CNO or an equivalent volume of saline 40 min before the start of recording. For statistical analyses, we used a RM-GLM to explore potential effects of recording layer (SO, SR, pyramidal, SLM), frequency (slow γ, fast γ), stimulus type (novel animal, novel object), and drug condition (vehicle, CNO), and results are presented in Table 1.

**Table 1:** *F* statistics and *p* values from the RM, GLM used to assess *Amigo2*-icreERT mice for effects of recording layer, frequency, drug condition, and stimulus type on oscillatory power

	Cre+		Cre–	
Source	*F* statistics	*p* value	*F* statistics	*p* value
Layer	25.31	**0.001**	24.04	**0.001**
Frequency	214.33	**0.001**	57.98	**0.001**
Drug	2.81	0.144	0.06	0.814
Stimulus	0.02	0.887	0.01	0.948
Layer × frequency	45.2	**0.001**	25.11	**0.001**
Layer × drug	0.25	0.86	0.41	0.748
Frequency × drug	0.3	0.606	0.77	0.422
Layer × frequency × drug	3.58	**0.034**	1.15	0.362
Layer × stimulus	0.08	0.973	0.01	0.999
Frequency × stimulus	0.69	0.439	0.8	0.411
Layer × frequency × stimulus	1.06	0.389	0.56	0.652
Drug × stimulus	0.36	0.571	0.3	0.606
Layer × drug × stimulus	0.89	0.465	0.55	0.654
Frequency × drug × stimulus	0.16	0.701	0.16	0.708
Layer × frequency × drug × stimulus	0.25	0.861	0.85	0.487

Results that reached statistical significance based on *p* = 0.05 are bolded and underlined.

In *Amigo2*-icreERT2*+* mice, we found significant main effects of layer (*F*_(1,3)_ = 25.31, *p *=* *0.000; *N* = 8) and frequency (*F*_(1,1)_ = 214.33, *p *=* *0.000; *N* = 8) as well as an interaction effect between layer and frequency (*F*_(1,3)_ = 45.20, *p *=* *0.000; *N* = 8). Additionally, we found a significant interaction among layer, frequency, and drug (*F*_(1,3)_ = 3.58, *p *=* *0.034; *N* = 8). By contrast, the RM-GLM revealed no main effect of stimulus (*F*_(1,1)_ = 0.02, *p *=* *0.887; *N* = 8) nor any significant interactions involving stimulus. Nevertheless, given our *a priori* hypothesis that inhibition of CA2 may alter γ oscillatory power in a stimulus-specific manner, we followed up by separating the data according to stimulus and compared γ power on either vehicle or CNO treatment for each stimulus exposure (Figs. 5, 6; and described below).

### Inhibition of CA2 decreases CA1 slow and fast γ power in a layer-specific manner but does not affect theta power

Because we found no main or interaction effects of stimulus type on γ oscillatory power in our experiments, we first collapsed data collected on investigation of social and object stimuli and asked how CNO versus vehicle treatment affected slow and fast γ power in each layer of CA1 (Figs. 3, 4). For slow γ oscillations, power was significantly less following CNO treatment than following vehicle treatment for recordings obtained from SO (*t*_(7)_ = 2.55, *p *=* *0.038; *N* = 8; Fig. 3*B*) and the pyramidal cell layer (*t*_(7)_ = 3.09, *p *=* *0.018; *N* = 8; Fig. 3*A*,*C*) of CA1, but not in recordings obtained from SR (*t*_(6)_ = 1.19, *p *=* *0.278; *N* = 7; Fig. 3*D*) or SLM (*t*_(6)_ = 0.34, *p *=* *0.745; *N* = 7; Fig. 3*E*). For fast γ oscillations, power was significantly less following CNO treatment than following vehicle treatment for recordings obtained from SO (*t*_(7)_ = 4.55, *p *=* *0.003; *N* = 8; Fig. 3*B*), the pyramidal cell layer (*t*_(7)_ = 7.36, *p *=* *0.0002; *N* = 8; Fig. 3*C*) and SR *t*_(6)_ = 3.35, *p *=* *0.015; *N* = 7; Fig. 3*D*) but not for recordings obtained from SLM (*t*_(6)_ = 1.90, *p *=* *0.107; *N* = 7; Fig. 3*E*).

We also measured theta powers and found no significant difference in theta power between CNO or vehicle treatment in *Amigo2*-icreERT2*+* for recordings obtained from SO (*t*_(7)_ = 1.45, *p *=* *0.192; Fig. 3*B*), the pyramidal cell layer (*t*_(7)_ = 0.466, *p *=* *0.655; Fig. 3*C*), SR (*t*_(6)_ = 0.999, *p *=* *0.356; Fig. 3*D*), or SLM (*t*_(6)_ = 1.52, *p *=* *0.180; Fig. 3*E*).

In control *Amigo2*-icreERT2– mice, we found a significant main effect of layer (*F*_(1,3)_ = 24.04, *p *=* *0.000; *N* = 7) and frequency (*F*_(1,1)_ = 57.98, *p *=* *0.001; *N* = 7), as well as an interaction effect between layer and frequency (*F*_(1,3)_ = 25.11, *p *=* *0.001; *N* = 7). We found no main effect of stimulus type or drug and no interactions involving stimulus type or drug (Table 1). Despite the absence of main or interaction effects of drug in *Amigo2*-icreERT2– mice, we compared each of slow and fast γ powers for each layer of CA1 following versus vehicle treatment because identical analyses were performed in *Amigo2*-icreERT2*+* mice.

For *Amigo2*-icreERT2– animals, slow γ power was not significantly different following CNO or vehicle treatment for recordings obtained from SO (*t*_(5)_ = 0.74, *p *=* *0.493; *N* = 6; Fig. 4*B*), the pyramidal cell layer (*t*_(6)_ = 0.33, *p *=* *0.756; *N* = 7; Fig. 4*A*,*C*), SR (*t*_(6)_ = 0.49, *p *=* *0.643; *N* = 7; Fig. 4*D*), or SLM (*t*_(6)_ = 1.26, *p *=* *0.253; *N* = 7; Fig. 4*E*). Similarly, fast γ power was not significantly different following CNO or vehicle treatment for recordings obtained from SO (*t*_(5)_ = 0.91, *p *=* *0.405; *N* = 6; Fig. 4*B*), the pyramidal cell layer (*t*_(6)_ = 0.96, *p *=* *0.373; *N* = 7; Fig. 4*C*), SR (*t*_(6)_ = 0.59, *p *=* *0.579; *N* = 7; Fig. 4*D*), or SLM (*t*_(6)_ = 67, *p *=* *0.527; *N* = 7; Fig. 4*E*).

Theta power was also not significantly different between CNO or vehicle treatment in *Amigo2*-icreERT2– mice for recordings obtained from SO (*t*_(5)_ = 1.16, *p *=* *0.299; Fig. 4*B*), the pyramidal cell layer (*t*_(6)_ = 0.351, *p *=* *0.737; Fig. 4*C*), SR (*t*_(6)_ = 0.438, *p *=* *0.677; Fig. 4*D*) or SLM (*t*_(6)_ = 0.226, *p *=* *0.829; Fig. 4*E*).

We also compared mean velocity of the animals during the times used for LFP analysis for each treatment and stimulus presentation and found no significant differences in locomotor velocity for either *Amigo2*-icreERT2*+* animals (main effect of stimulus: *F*_(1,7)_ = 0.000629, *p *=* *0.981; main effect of treatment: *F*_(1,7)_ = 1.083, *p *=* *0.333) or *Amigo2*-icreERT2– animals (main effect of stimulus: *F*_(1,6)_ = 0.1282, *p *=* *0.733; main effect of treatment: *F*_(1,6)_ = 0.02,376, *p *=* *0.883).

### Inhibition of CA2 pyramidal cells reduces slow γ power on social but not object stimulation

Our *a priori* hypothesis before beginning this study was that inhibition of CA2 may alter γ oscillatory power in a stimulus-specific manner. Therefore, as a follow-up analysis to that presented in Figures 3, 4, we separated γ oscillatory power data according to stimulus and compared slow and fast γ power upon either vehicle or CNO treatment for each type of stimulus exposure.

For *Amigo2*-icreERT2*+* animals, we found that slow γ power was significantly reduced in the pyramidal cell layer upon CNO treatment compared with vehicle treatment selectively during novel animal investigation; slow γ power was not significantly reduced following CNO treatment during novel object investigation but was significantly reduced during novel animal investigation (main effect of treatment: *F*_(1,7)_ = 9.5, *p *=* *0.0178; main effect of stimulus: *F*_(1,7)_ = 2.357; *p *=* *0.1686; interaction: *F*_(1,7)_ = 1.099, *p *=* *0.3293; novel animal: *p *=* *0.0136, novel object: *p *=**0.1063). In the SO layer, slow γ power was overall significantly reduced following CNO treatment compared with vehicle treatment but each stimulus alone did not significantly reduce slow γ power (main effect of treatment: *F*_(1,7)_ = 6.477, *p *=* *0.0384; main effect of stimulus: *F*_(1,7)_ = 0.5069; *p *=* *0.4995; interaction: *F*_(1,7)_ = 0.4712, *p *=* *0.5145; novel animal: *p *=* *0.1015, novel object: *p *=* *0.3913). Slow γ power was not significantly reduced by CNO in either SR (main effect of treatment: *F*_(1,6)_ = 1.416, *p *=* *0.2790; main effect of stimulus: *F*_(1,6)_ = 0.08,567; *p *=* *0.7796; interaction: *F*_(1,6)_ = 0.432, *p *=* *0.5354) or SLM (main effect of treatment: *F*_(1,6)_ = 0.1542, *p *=* *0.71 081; main effect of stimulus: *F*_(1,6)_ = 0.02,751; *p *=* *0.837; interaction: *F*_(1,6)_ = 0.2365, *p *=* *0.6440).

Similar to slow γ power in the pyramidal cell layer, among *Amigo2*-icreERT2*+* animals, fast γ power in the pyramidal cell layer was also significantly reduced following CNO treatment. Interestingly though, whereas slow γ power reduction was selective for social stimulus exposure, fast γ power in the pyramidal cell layer was significantly reduced during both novel social stimulus and novel object stimulus exposure (main effect of treatment: *F*_(1,7)_ = 55.47, *p *=* *0.0001; main effect of stimulus: *F*_(1,7)_ = 0.003674; *p *=* *0.9534; interaction: *F*_(1,7)_ = 0.9945, *p *=* *0.3519; novel animal: *p *=* *0.0109, novel object: *p *=* *0.0021). In the SO and SR layers, fast γ power was overall significantly reduced following CNO treatment compared with vehicle treatment (SO: main effect of treatment: *F*_(1,7)_ = 20.82, *p *=* *0.0026; main effect of stimulus: *F*_(1,7)_ = 0.001639, *p *=* *0.9688; interaction: *F*_(1,7)_ = 0.0263, *p *=* *0.8758; SR: main effect of treatment: *F*_(1,6)_ = 11.2, *p *= 0.0155; main effect of stimulus: *F*_(1,6)_ = 0.1965, *p *=* *0.6731; interaction: *F*_(1,6)_ = 0.3173, *p *=* *0.5936) but each stimulus alone did not significantly reduce slow γ power (SO: novel animal: *p *=* *0.1303; novel object: *p *=* *0.1813; SR: novel animal: *p *=* *0.6322; novel object: *p *=* *0.2553). Fast γ power was not significantly reduced by CNO in SLM (main effect of treatment: *F*_(1,6)_ = 3.574, *p *=* *0.1076; main effect of stimulus: *F*_(1,6)_ = 1.022, *p *=* *0.3511; interaction: *F*_(1,6)_ = 4.355, *p *=* *0.0820).

Among *Amigo2*-icreERT2– animals (Fig. 6), we found no significant difference in slow γ or fast γ powers on either CNO or vehicle administration for recordings obtained from SO (slow γ: main effect of treatment: *F*_(1,5)_ = 0.5475, *p *=* *0.4926; main effect of stimulus: *F*_(1,5)_ = 0.01,439 *p *=* *0.9092; fast γ: main effect of treatment: *F*_(1,5)_ = 0.8265, *p *=* *0.4050; main effect of stimulus: *F*_(1,5)_ = 2.188, *p *=* *0.1992), the pyramidal cell layer (slow γ: main effect of treatment: *F*_(1,6)_ = 0.1057, *p *=* *0.7562; main effect of stimulus: *F*_(1,6)_ = 0.02,277, *p *=* *0.8850; fast γ: main effect of treatment: *F*_(1,6)_ = 0.9247, *p *=* *0.3734; main effect of stimulus: *F*_(1,6)_ = 1.999, *p *=* *0.2072), SR (slow γ: main effect of treatment: *F*_(1,6)_ = 0.2309, *p *=* *0.6479; main effect of stimulus: *F*_(1,6)_ = 0.1713, *p *=* *0.6933; fast γ: main effect of treatment: *F*_(1,6)_ = 0.3515, *p *=* *0.5749; main effect of stimulus: *F*_(1,6)_ = 1.321, *p *=* *0.2941) or SLM (slow γ: main effect of treatment: *F*_(1,6)_ = 1.616, *p *=* *0.2507; main effect of stimulus: *F*_(1,6)_ = 2273, *p *=* *0.6504; fast γ: main effect of treatment: *F*_(1,6)_ = 0.4432, *p *=* *0.5303; main effect of stimulus: *F*_(1,6)_ = 0.2385, *p *=* *0.6426).

### CA2 pyramidal cell inhibition does not affect investigation time

**Figure 7.**
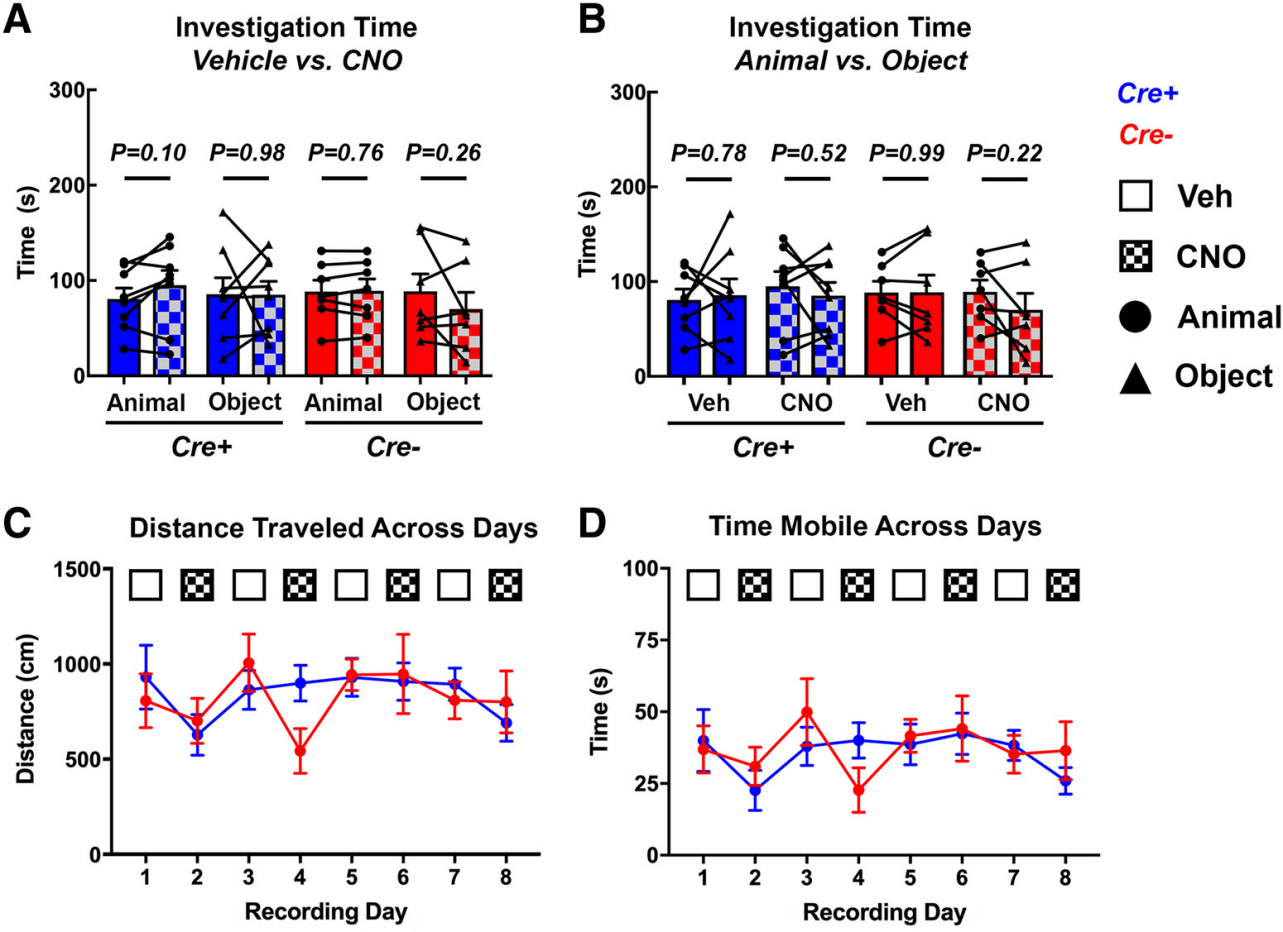
Effect of CNO on stimulus investigation time and activity level. ***A***, Time subject mice spent investigating different stimuli following administration of CNO or vehicle. CNO did not significantly affect the amount of time *Amigo2*-icreERT2+ or *Amigo2*-icreERT2– mice spent investigating animals or objects. ***B***, Comparison of time spent investigating animals versus objects following either vehicle or CNO administration. Neither *Amigo2*-icreERT2+ nor *Amigo2*-icreERT2– mice exhibited a preference for one stimulus type over the other following vehicle or CNO administration. ***C***, ***D***, Mean distance traveled (***C***) and time spent mobile (***D***) measured across recording days for *Amigo2*-icreERT2+ (blue line) and *Amigo2*-icreERT2– (red line) mice. Mobile periods were based on a 7-cm/s movement threshold with speeds greater than the threshold considered periods of mobility. Days on which CNO was administered are indicated by hash-marked bars or boxes, and days on which vehicle was administered are indicated by solid white or color bars or boxes. *Amigo2*-icreERT2+ cohorts are represented by red lines and bars and *Amigo2*-icreERT– cohorts are represented by blue lines and bars. For ***A***, ***B***, data points represent individual subject values and bars represent group averages. Circles indicate when an animal was used as the stimulus and triangles indicate when the stimulus was an object. Error bars indicate the SEM.

Based on our findings of reduced slow and fast γ power on acute inhibition of CA2 pyramidal cells during investigation of novel stimuli, we asked whether the effects of CNO observed on neuronal oscillations were accompanied by changes in behavior that may have been responsible for changes in neuronal activity. First, we assessed whether CNO administration alone caused changes in the time subjects spent investigating the different types of stimuli. In *Amigo2*-icreERT2+ mice, CNO did not significantly affect the amount of time subjects spent investigating novel animals (*N* = 8; *t*_(7)_ = 1.91, *p *=* *0.098; Fig. 7*A*) or novel objects (*N* = 8; *t*_(7)_ = 0.02, *p *=* *0.981). Similarly, in control *Amigo2*-icreERT2– mice, CNO did not cause a significant change in the amount of time subjects spent investigating novel animals (*N* = 7; *t*_(6)_ = 0.32, *p *=* *0.761; Fig. 7*A*) or novel objects (*N* = 7; *t*_(6)_ = 1.25, *p *=* *0.257). Additionally, we assessed whether subjects showed a preference for either the novel animal or novel object stimulus following either vehicle or CNO administration based on time spent investigating the different stimulus types. *Amigo2*-icreERT2+ mice did not exhibit a preference for either stimulus type following vehicle (*N* = 8; *t*_(7)_ = 0.29, *p *=* *0.784; Fig. 7*B*) or CNO (*N* = 8; *t*_(7)_ = 0.67, *p *=* *0.521) administration. Similarly, control *Amigo2*-icreERT2– mice did not show a preference for either stimulus type following vehicle (*N* = 7; *t*_(6)_ = 0.02, *p *=* *0.985; Fig. 7*B*) or CNO (*N* = 7; *t*_(6)_ = 1.37, *p *=* *0.221) administration. Additionally, we measured and found no difference in the average distance traveled across experimental days by *Amigo2*-icreERT2+ (*F*_(3.421,23.95)_ = 2.141, *p = *0.1149; Fig. 7*C*, blue line) or *Amigo2*-icreERT2– mice (*F*_(3.027,18.16)_ = 1.772, *p = *0.1880; Fig. 7*C*, red line). Finally, we found no difference in the average time spent mice mobile across experimental days by *Amigo2*-icreERT2+ (*F*_(3.459,24.21)_ = 2.082, *p = *0.1219; Fig. 7*D*, blue line) or *Amigo2*-icreERT2– mice (*F*_(3.131,18.78)_ = 1.528, *p *=* *0.2394; Fig. 7*D*, red line). Therefore, the changes in slow and fast γ powers on acute inhibition of CA2 are not likely to be due to changes in behavior.

## Discussion

In this study, we recorded LFPs from across CA1 layers in adult, male *Amigo2-*icreERT2 mice and examined neuronal oscillations in the theta, slow γ and fast γ frequency ranges while subjects freely explored novel juvenile conspecifics or novel inanimate objects that were confined to a single position in space. We then chemogenetically inhibited CA2 pyramidal cells using hM4Di to determine whether CA2 contributes to the organization of theta, slow γ or fast γ oscillations in CA1 during investigation of novel stimuli. We found that acute inhibition of CA2 pyramidal cells reduced γ power in a layer- and frequency-dependent manner, with effects limited to the dendritic domains targeted by CA2 axons. In addition, analysis of changes in γ power by each type of stimulus revealed a stimulus-specific effect of silencing CA2 on γ oscillatory power; CA2 inhibition decreased slow γ power in the pyramidal cell layer during investigation of novel animals but not novel objects. Interestingly, CA2 inhibition decreased fast γ power in the pyramidal cell layer during both novel animal and novel object investigation. No significant change in γ power was detected in SLM, the only layer not targeted by CA2 neurons. In addition, we observed no significant change in theta oscillatory power on CA2 inhibition. These findings reveal three novel points about the role of CA2 in organizing hippocampal oscillatory networks.

First, CA2 modulates γ activity in the SR and SO dendritic layers of CA1 in addition to the pyramidal cell layer but does not affect SLM. The generation of independent, layer-specific γ oscillations in CA1 has previously been shown to depend on selective axonal targeting from upstream regions that modulate firing of CA1 GABAergic interneurons (Lasztóczi and Klausberger, 2014). CA2 pyramidal cells provide feed-forward inhibition as well as direct excitation of CA1 neurons (Valero et al., 2015; Boehringer et al., 2017), enabling it to modulate the balance of synaptic excitation and inhibition, a key feature of γ oscillations. In addition to engaging interneurons within CA1, CA2 neurons target local inhibitory basket cells that project to CA1 (Mercer et al., 2012), providing another source of CA2-driven inhibition in CA1. Additionally, shifts in CA1 output have been shown to reflect discrete excitatory events generated by CA3 Schaffer collateral input at the γ frequency, which serves as an accurate readout of the presynaptic population’s activity level (Fernández-Ruiz et al., 2012). Thus, the observation that CA2 inhibition caused a selective reduction in γ power in the dendritic domains targeted by CA2 axons suggests that CA2, and not some other CA1-projecting region, is responsible for the observed modulation of γ power. These data support the idea that γ oscillations facilitate communication between CA2 and CA1 in a manner similar to the CA3-CA1 interface.

Second, these experiments suggest that in addition to regulating slow γ oscillations, CA2 neuronal activity may serve to amplify fast γ oscillations in CA1. Slow γ oscillations in CA1 have previously been shown to rely on CA3 Schaffer collateral input (Middleton and McHugh, 2016). In addition, CA2 has been shown to contribute to the generation of slow γ oscillations in the pyramidal cell layer of CA1 while subjects traverse an open field with no stimulus present (Alexander et al., 2018). Here, we confirm and extend those findings by demonstrating that the CA1 layers where slow γ oscillations are affected by CA2 silencing match the layers to which CA2 neurons project. Fast γ oscillations, by contrast, have been shown to rely on medial EC input to dentate gyrus and SLM (Bragin et al., 1995), and CA2 inhibition was previously not shown to decrease fast γ while subject traversed an open field with no stimuli present (Alexander et al., 2018). By contrast, our findings demonstrate a role for CA2 activity in fast γ oscillations in CA1 during immobile investigation of novel stimuli in that silencing of CA2 output decreased fast γ power in SO, SR and the pyramidal cell layer. One possible explanation for this finding is that CA2 activity may normally facilitate fast γ oscillation propagation within the hippocampal circuitry during investigation of novel stimuli (animal, object, or otherwise), and bilateral silencing of CA2 may remove this faciliatory role of CA2.

Third, we provide evidence that slow γ oscillations are decreased in response to CA2 silencing during investigation of novel social stimuli but not novel object stimuli. CA2 has been shown to be required for social recognition memory (Hitti and Siegelbaum, 2014), but whether CA2 neurons coordinate their activity with CA1 when processing stimuli containing socially relevant information remained unexplored. We predicted that if CA2 is preferentially involved in processing information about socially relevant stimuli, then CA2 pyramidal cells may organize rhythmic activity in CA1 only during investigation of an animal and not an object such that silencing of CA2 activity would impact oscillatory activity preferentially during investigation of social stimuli. Further, based on the layer-specificity of CA2 axons within CA1, we expected that any altered oscillatory activity would be observed selectively in CA2-projection layers. Indeed, we found that γ oscillatory power recorded from a CA2-projection layer was affected by CA2 inhibition during social investigation.

Given the previous finding that chemogenetic modulation of CA2 neuronal activity affects slow γ power in the CA1 pyramidal cell layer (Alexander et al., 2018), we favored the hypothesis that slow γ power but not high γ power would be decreased by CA2 silencing during novel animal investigation. Accordingly, we found that slow γ power was reduced on CA2 inhibition. Somewhat surprisingly though, we also found that fast γ power was reduced on CA2 inhibition and it was reduced during both novel animal and novel object investigation. CA2 neuronal activity patterns have been shown to change (i.e., place cell remapping) in response to novel conspecifics and to novel objects (Alexander et al., 2016), and CA2 neurons have been shown to respond to novelty even when there is no social component involved in the task (Wintzer et al., 2014; Lu et al., 2015), so it is not surprising that novel object investigation as well as novel animal investigation would engage CA2 neuronal activity. Possibly, slow γ oscillations originating from activity of CA2 and CA3 neurons are engaged by social investigation, whereas fast γ oscillations originating from activity of medial EC neurons and propagated by way of the dentate gyrus to hippocampus in response to investigation of any novel stimulus (Kemere et al., 2013; Zheng et al., 2016), are facilitated by the activity of CA2 neurons.

Based on our physiological findings, we asked whether the observed reductions in γ power were accompanied by changes in behavior that may have produced the changes in neuronal oscillations. We measured time spent investigating the different types of stimuli following treatment with CNO or vehicle and found no difference in the amount of time mice spent investigating either stimulus type following either treatment. Because we were interested in the role of CA2 in hippocampal oscillatory networks and the electrophysiological effects of targeted CA2 manipulation, we selected a behavior that did not require behavioral training. For this reason, the lack of a significant effect of CA2 inhibition on stimulus investigation times is not surprising, as we used a passive exposure paradigm in which subjects could freely explore the environment of their own volition, with each session separated by 24 h. In contrast, social recognition paradigms typically measure investigation time of social stimuli on repeated exposures taking place over the course of minutes to hours (Hitti and Siegelbaum, 2014; Smith et al., 2016). These differences in experimental design do not necessarily suggest that the electrophysiological effects described here are not relevant to behavior, but that a behavioral paradigm explicitly designed to assess changes in memory would be required to reveal their behavioral relevance. Further, the lack of behavioral changes in our study makes the interpretation of electrophysiological data more straightforward, as differences in behavior can also cause changes in oscillatory activity and confound whether observed effects are due to experimental manipulation or the different behaviors performed.

In addition, we note that the dose of CNO we used (3 mg/kg) was lower than that used in previous study of CA2 and social memory (10 mg/kg; Meira et al., 2018). However, a lower dose of 5 mg/kg CNO was previously shown to silence CA2-driven responses in CA1 by >95% (Alexander et al., 2018), and a dose of 4 mg/kg CNO has been shown to effectively inhibit social memory by inhibiting ventral CA3 neuron activity (Chiang et al., 2018). Therefore, although not examined in the current study, we would expect that the CNO dose used in the current study would impair social recognition memory and is appropriate for study of neuronal activity patterns that may be involved in social memory.

In conclusion, we found that CA2 neuronal activity contributes to slow and fast γ oscillations in CA1 in a dendritic layer-dependent manner. Slow γ oscillations were preferentially impacted by CA2 silencing during novel animal investigation over novel object investigation, whereas fast γ oscillations were affected by CA2 silencing during either novel animal or novel object investigation. These findings support a role of CA2 neuronal activity in hippocampal γ oscillations and processing of social and contextual information.

## Synthesis

Reviewing Editor: Thomas McHugh, RIKEN Center for Brain Science Decisions are customarily a result of the Reviewing Editor and the peer reviewers coming together and discussing their recommendations until a consensus is reached. When revisions are invited, a fact-based synthesis statement explaining their decision and outlining what is needed to prepare a revision will be listed below. The following reviewer(s) agreed to reveal their identity: NONE.

The authors have addressed the concerns raised by the reviewers at the Journal of Neuroscience and is ready for publication at eNeuro.
